# SafeDrive: Hybrid Recommendation System Architecture for Early Safety Predication Using Internet of Vehicles

**DOI:** 10.3390/s21113893

**Published:** 2021-06-04

**Authors:** Rayan Nouh, Madhusudan Singh, Dhananjay Singh

**Affiliations:** 1 Institute of Consulting and Research Studies, Umm Al-Qura University, Mecca 18135, Saudi Arabia; rmnooh@uqu.edu.sa; 2 Endicott College of International Studies, Woosong University, Daejeon 300718, Korea; 3 Department of Electronics Engineering, Hankuk University of Foreign Studies, Yongin 17035, Korea; dsingh@hufs.ac.kr

**Keywords:** ITS, IoV, deep learning, recommender system, driving behavior and road safety

## Abstract

The Internet of vehicles (IoV) is a rapidly emerging technological evolution of Intelligent Transportation System (ITS). This paper proposes SafeDrive, a dynamic driver profile (DDP) using a hybrid recommendation system. DDP is a set of functional modules, to analyses individual driver’s behaviors, using prior violation and accident records, to identify driving risk patterns. In this paper, we have considered three synthetic data-sets for 1500 drivers based on their profile information, risk parameters information, and risk likelihood. In addition, we have also considered the driver’s historical violation/accident data-set records based on four risk-score levels such as high-risk, medium-risk, low-risk, and no-risk to predict current and future driver risk scores. Several error calculation methods have been applied in this study to analyze our proposed hybrid recommendation systems’ performance to classify the driver’s data with higher accuracy based on various criteria. The evaluated results help to improve the driving behavior and broadcast early warning alarm to the other vehicles in IoV environment for the overall road safety. Moreover, the propoed model helps to provide a safe and predicted environment for vehicles, pedestrians, and road objects, with the help of regular monitoring of vehicle motion, driver behavior, and road conditions. It also enables accurate prediction of accidents beforehand, and also minimizes the complexity of on-road vehicles and latency due to fog/cloud computing servers.

## 1. Introduction

The Internet of vehicles (IoVs) and the recent technologies fused with the intelligent vehicles, ensure road safety by preventing and detecting road accidents accurately. Recent technologies fused with intelligent vehicles ensure road safety by preventing and detecting road accidents accurately [1]. A dynamic personalized analysis of driving behavior is possible when traffic data is processed with advanced AI technology. According to WHO, 1.35 million people die each year as a consequence of road accidents and a large quantity of the population suffer from road accident injuries, which affect economic and human losses. Approximately 85% of the road traffic accidents happen due to human error [2]. A recent study shows that around 50 million people suffer from non-fatal injuries, with a large number of them experience a disability as a consequence of their injury. Therefore, driver behavior is the major contributing factor in road crashes in the world. In the broad domain of accident prevention and road safety, driver behavior analysis is a main focus since a large number of accidents are due to drowsiness of the drivers [3]. The current smart transportation system uses an alert generation module to send immediate alerts to the corresponding vehicles in the monitoring system. The personalized recommendation system is one of the methods to provide a safe transportation system based on road traffic crashes and violations on the record of the driver, giving recommendations along with increased personal awareness to enhance the driving behavior. In behavior detection, steering position, vehicle position, driver’s eye/face, and physiological measurement play pivotal roles. The recommendations of driver feedback are based on driver historical violations and accident records to predict current and future driver risk scores using the Saudi Traffic Points System Regulation (STPSR) system [4]. In the analysis of driver behavior, it is also necessary to consider the traffic characteristics and junction characteristics to achieve better accuracy. It has been observed that numerous researchers are working on road safety solutions which aim to ensure a safe transport system. The majority of the research in ITS has been in exploiting advances in the fields of electronic systems to estimate the various responsible parameters for causing on-road accidents and traffic congestion. The primary shortcoming of the electronic system is that it has to analyses the results from the physical factors such as limitations in communication infrastructure, environmental, and surrounding conditions. Currently, machine learning is gaining attention to play a significant role in the management of numerous tasks such as in traffic data study, analysis, and classifications, which helps to personalize recommendations of various contents that can become adding value assets for most of the ITS by analyzing the historical driving behavior records.

**Figure 1:**
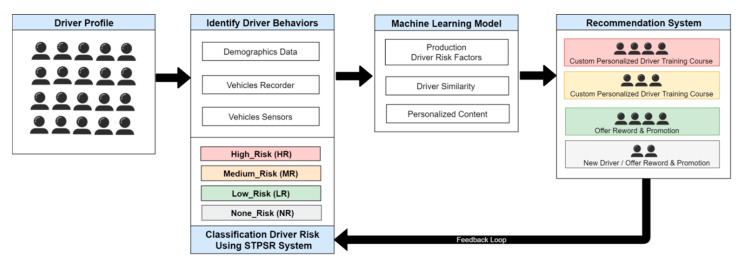
DDP based on hybrid recommendation system architecture.

In this study, we developed a hybrid recommendation system for DDP data management architecture to predict the future driving risk of crash-involved drivers shown in Figure 1. For this study, we have considered the STPSR dataset to predict the driving behavior based on the driving risk, and identify risky driving factors. With the help of a reliable and explicable machine learning method, we have predicted the high-risk (HR), medium-risk (MR), low-risk (LR), and no-risk drivers for further driving. The number of points calculated for every violation/accident of traffic regulation risk likelihood based on the risk matrix classifies the parameter using the STPSR datasets. Moreover, the paper describes the potential of the risk modeling of the drivers, provides feedback to them, monitors behavior, and focuses on the driver risk behavior based on accident severity and traffic violations using rule-based classification.

1. We developed a SmartDrive recommendation system for drivers’ driving skill management and prediction using the concept of the machine learning model.
2. We propose a dynamic driver profile (DDP) approach to analyse individual drivers’ behaviors to identify a driver’s risk pattern based on four risk-score levels such as high-risk, medium-risk, low-risk, and no-risk.
3. We show improvements in the prediction accuracy of the recommendations are evaluated on the three performance errors: mean squared error (MSE), mean absolute percentage error (MAPE), and mean absolute error (MAE) for the Saudi Arabian traffic accident and violation data to identify trends, recognize underlying factors influencing traffic accidents, and provide recommendations and key findings and insights.

The rest of the paper is organized as follows. In Section 2, we have summarized the related work and our contribution. In Section 3, we provide an overview of the proposed hybrid recommendation system architecture in detail. Section 4 presents the deep learning modeling and data classification algorithms for DDP. Section 5 provides the performance analysis and use case implementation of the proposed method with the real-world application in the Saudi Arabian transportation system, and finally we have concluded our work in Section 6.

## 2. Related Work

In order to improve ITS and future driving risk indexes, many researchers have presented a wide range of solutions, in which car crashes, head-on accidents, fires, and roll-on events can be accurately detected with the assist of on-board sensors that are deployed in the intelligent vehicles [5]. In the broad field of accident prevention and road safety, driver behavior analysis takes most of the focus since a large number of accidents are due to drowsiness of the drivers [6]. In the analysis of driver behavior, it is also necessary to consider the traffic characteristics and junction characteristics to achieve better accuracy [7]. Additionally, the current smart systems use alert generation modules in the monitoring systems to send immediate alerts the corresponding vehicles. In behavior detection, steering position, vehicle position, driver’s eye/face, and physiological measurement play pivotal roles [8]. On the other hand, trajectory prediction through the past sequences and current behavior of the vehicle and driver correspondingly pull much attention [9]. The multi-modal Kalman filter is used for trajectory prediction that works upon the extended Kalman filter. The proposed Kalman filter model mainly uses three different parameters, vehicle velocity, position, and distance of the vehicle from the intersection, to construct the state vector matrix [10]. In the case of road safety, the trajectory must be predicted as early as possible to avoid collisions and accidents, thus long-short-term-memory (LSTM) based trajectory prediction is ineffectual. The convolutional neural network (CNN) for motion detection and the recurrent neural networks (RNN) for movement planning have been utilized for vehicle surrounding and movement prediction for autonomous vehicles during the multi-lane turn crossings. The autonomous vehicle updates its motion based on the trajectories of the surrounding vehicles to prevent accidents and to improve road safety [11]. Also, the lane-change intention, which is an important aspect of multi-lane roads, was not considered. This degrades the prediction accuracy. The surrounded vehicle trajectory is only predicted to avoid accidents and collisions, but surrounding vehicles’ behavior and maneuvers are the major factors for road safety. The safety is assured only for subject vehicle and the other vehicles and pedestrians are still in unsafe condition. Specifically, LSTM-RNN are useful in prediction of the trajectory, and the future trajectory of the vehicle is predicted from this value. The trichotomyAdaboost (AdaBoost-SO) approach was used for accident risk prediction [12]. The data considered in this work are related to the vehicle and its direction on the road. An accident detection-based IoT system is proposed to report the accidents earlier in smart city environments where the vehicle sensors collect and process the data with the help of on-board units (OBUs). For each sensor reading, a threshold value is pre-defined to detect the abnormalities in the system [13]. DeepCrash is the deep learning-based accident detection and alert generation system that uses a densely connected convolutional neural network (DenseNet) for processing the vehicle data in the cloud environment. Based on the classification report, the alert is generated and sent to the vehicles [14]. An intelligent and smart IoV system demands early prediction and warning of accidents to improve road safety. Firstly, challenging the methods of processing huge data in OBUs degrades the accuracy and increases complexity; secondly, cloud-based data management and detection increases latency for alert generation; thirdly, involvement of a high false alarm rate increases collisions; and finally, some early warning systems have poor accuracy [15]. A fuzzy inference system-ased driver monitoring system (FDMS) has considered two individual fuzzy models for decision making in safe driving. The first fuzzy model takes the vehicles’ environment temperature, noise level, and driver’s heart rate as inputs. The second fuzzy model takes respiratory rate of the driver to compute the driver’s situational awareness (DSA). With the help of these two FDMS, the final decision on the driver’s behavior is made based on whether it is a normal situation, bad situation, or worst situation [16]. An automated accident detection and classification system has been focused on the emergency medical service (EMSs) like ambulances, provisioning rescue operations by improving the prediction of road conditions. Where the smartphone-assisted sensors are used to collect data from the vehicles and environment. The system was tested using naïve Bayes, Gaussian mixture model (GMM) and decision tree (DT) algorithms [17]. Artificial intelligence-based vehicle behavior anticipation mechanisms have been discussed in [18] where a hybrid vehicle trajectory prediction methodology has been developed using a long short term memory (LSTM)-based trajectory prediction model which has demonstrated the use of maneuver-based attributes such as lane changing for trajectory prediction.

This paper proposes a LSTM-based motion detection method. Towards that aim, a motion planner-based model predictive control (MPC) was designed to support multi-lane environments. Here, the autonomous vehicle updates its motion based on the trajectories of the surrounding vehicles to prevent accidents and to improve road safety. The dataset considered is related to the multi-lane turn intersections data. This data is classified by LSTM-RNN into left-lane, right-lane, and lane-keeping. Considering the predicted trajectory, travel time and motion of the autonomous vehicle is planned. Therefore, the LSTM is generally a complex network that consumes a large amount of time. In the case of road safety, the trajectory must be predicted as early as possible to avoid collisions and accidents. Thus, LSTM-based trajectory prediction is ineffectual. Here, lane-change intention, which is an important aspect of multi-lane roads, is not considered. This degrades the prediction accuracy. The surrounded vehicle trajectory is only predicted to avoid accidents and collisions, but the surrounding vehicles’ behavior and maneuvers are the major factors for road safety.

## 3. Hybrid Recommendation System Architecture

**Figure 2:**
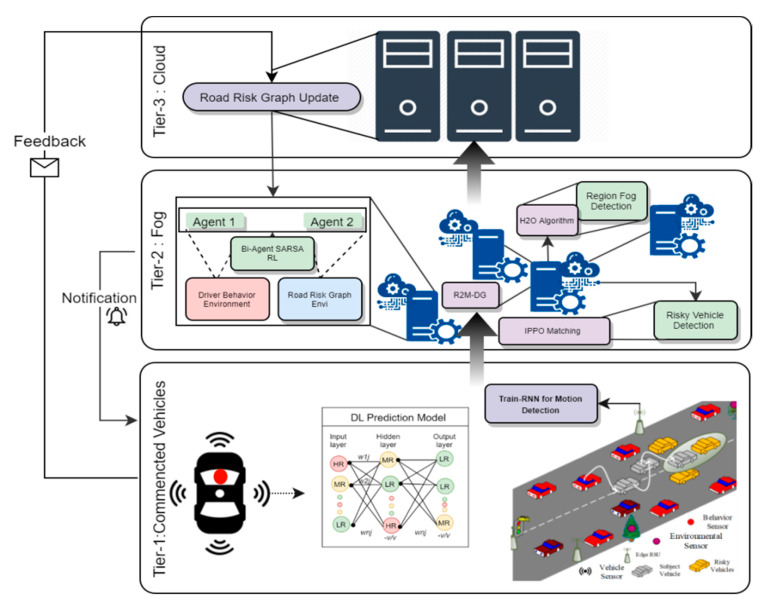
SafeDrive architecture for three-tier alert system.

A recommendation system gives us opportunities to provide a set of services and systems. The proposed hybrid recommendation system architecture aims to tackle the challenges in preventing road accidents and generating early warning alerts as a part of road safety in the connected vehicles environment. The proposed SafeDrive architecture platform equipped with behavioral sensors for the driver’s driving prediction mechanism process are shown in Figure 2, where the collected data from sensors is transferred to the upper layers through communication technologies. It is divided in three tiers:

- Tier 1 (Connected Vehicles): This tier includes the intelligent connected vehicles which have in-built sensors such as speed, acceleration etc. It includes the on board unit (OBU) and edge road side units (E-RSUs) to enhance the processing speed.
- Tier 2 (Fog Computing): This tier includes distributed fog nodes, each responsible for monitoring and handling separate regions in Tier 1. The fog nodes have processing abilities higher than E-RSUs.
- Tier 3 (Cloud Computing): This is the uppermost tier and includes a centralized cloud server which maintains the continuous monitoring information generated from the Tier 1 and Tier 2 devices.

All three tiers work together to prevent accidents and generate the early warning alerts to the vehicles for providing safety services. For that, the Safe-Drive model requires multiple contributions, such as: deep vehicle motion prediction, dependency graph–road risk map construction, reinforced driver behavior analysis, and optimal alert generation for road safety. Deep vehicle motion prediction analysis depends upon three main attribute sets: lane change information (maneuver based), nearby vehicle report (interactive aware), and past instances of the vehicle. All three sets are learned individually by the tri-set independent recurrent neural network (TriIn-RNN) model. The dependency graph–road risk map construction process is based on road conditions and the structure, where each fog node constructs a road risk map (R2M) as the dependency graph (DG) for its underlying region, which is determined by Bayesian probability. Reinforced driver behavior analysis is analyzed as the function of respiratory rate, heart rate, eye closure rate, EEG factor, etc. From these features, the first agent learns the driver behavior. Another agent learns road conditions from R2R-DG, which are constructed and updated effectually as normal, bad, or worst conditions. The optimal alert generation for road safety process is based on the current state, which is determined as bad or worse, and then the fog node triggers the alert module to send alerts to the vehicles.

### 3.1. Hybrid Recommendation System

**Figure 3:**
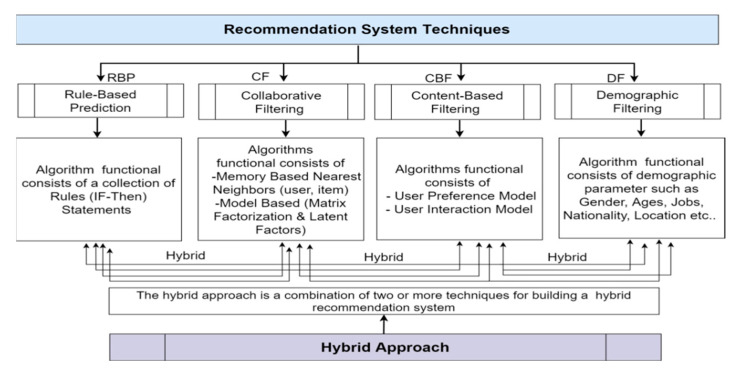
Hybrid approach based recommendation system techniques.

The safety is assured only for the subject vehicle and the other vehicles and pedestrians are still in an unsafe condition. Therefore, we have tried to overcome the current research limitations, such as to provide the correct information needed by a particular user so that the same user can choose the best driver or in specific fields. A dynamic driver provided recommendation system allows the user to choose the best vehicle trajectory based on the user’s required information. Based on the background studies, we have conceptualized a standard recommendation techniques in Figure 3, which is important to incorporate the information related to the user in the recommendation system. The method used is most relevant information per user, ignoring other information related to the user such as the weather, time, location, etc.

The dynamic information of user contacts has been used in previous research in relation to recommendation systems. These studies show that to provide recommendations, it is important to incorporate the information related to the user in the RS. A model of contextual filtering also has some limitations related to its application in a single dimension, as in the case of a user’s neighbor [19]. To overcome these deficiencies, we propose a personalized recommendation system that learns from driver behavior contents and identified risk factors using practical machine learning approaches.

### 3.2. Dynamic Driver Profile (DDP)

**Figure 4:**
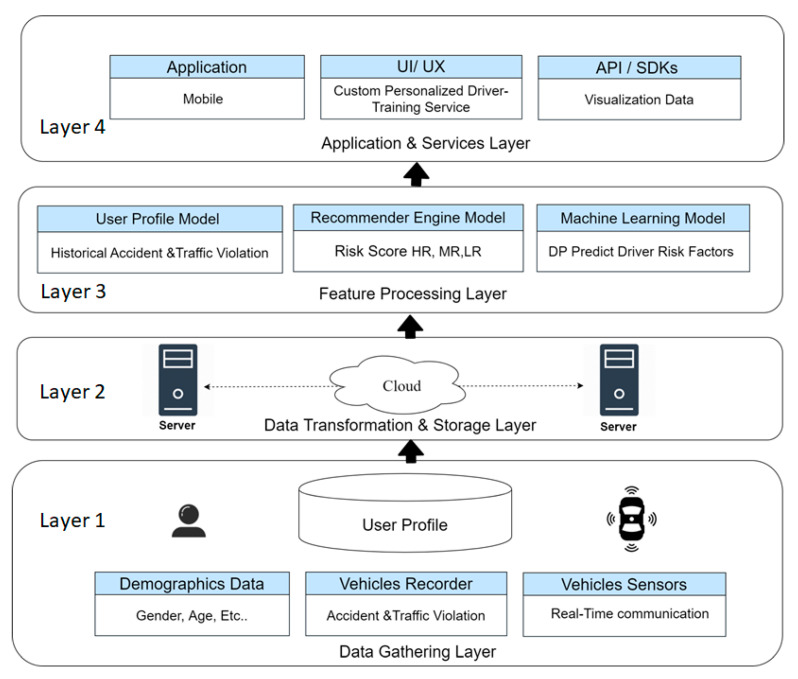
DDP—dynamic driver profile features.

The hybrid recommendation system is based on the concept of dynamic driver profile (DDP), which is an umbrella framework that provides a set of services and systems. We focused on preventing road accidents and generating early warning alerts as a part of road safety in connected vehicles. Towards this aim a novel four-tier alert DDP platform architecture was designed as shown in Figure 4, where the first layer (Layer 1) data gathering includes demographics, vehicle, accident, and violation records data. The second layer (Layer 2) cloud computing server includes transformation and integrated data multiple dataset sources. The third layer (Layer 3) data processing stage includes the three modules of user profile, recommender system, and machine learning techniques. Finally the fourth layer (Layer 4), the application and services layer, includes the API to custom personalized content, visualization data, and generate driver report to different stockholders such as government-to-consumer (G2C) individual and report, government-to-business (G2B) enterprise report, and government-to-government (G2G) report. In the DDP scenario, we can send routine safety messages to risky drivers with potentially high-risk driver behavior, which can be recommended learning content based on their risk driver’s behavior and request to attend driving lecture or pass exams and drivers can get usage/based insurance pay as you drive for the safety messages.

The transformation of the physical driver behaviors into a digital service offers possibilities for delivering benefits to users and increases the value for them in using the service. Therefore, these services provide benefits to all different stakeholders, such as citizens, businesses, and government. In the G2C, individual DDPs can issue a violations and accidents history record in addition to the driver record to give wider background about the car’s record in term of usage before the purchasing decision. For G2G/G2B, business government DDPs can issue a vehicle history record which gives the decision makers a solid background about their car’s condition, thus they can easily decide which car can stay with the fleet and which car must be replaced [20]. G2B for insurance companies through the vehicle history report DDP can provide a clear insight about each car to help the insurance companies to review their pricing list and distinguish between a car in good condition and one in bad condition. After one year of collecting the driver’s data we can offer a forecast report about the probability of violation or accident occurrence for each insure.

### 3.3. DDP Components

**Figure 5:**
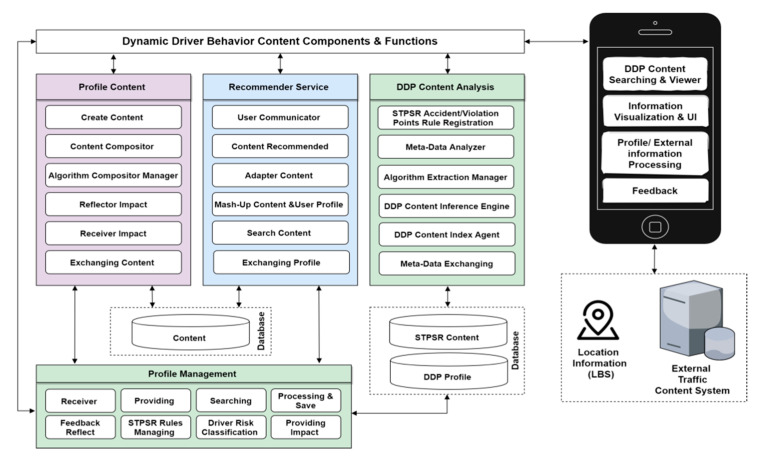
DDP components and functions.

The DDP components and functions are described in Figure 5, which provides an overview of the key part of a DDP that provide personalized content to enhance the driver behavior and rode safety. The design of a major intelligent recommendation platform has to provide a set of services and identifies the major functional components, while the relationships between these components were organized into the following four-fold system DDP content components and functions:

- User profile contents processing and management;
- Recommendation processing and services;
- Driver behaviors analysis processing; and
- Profile management and processing.

These four systems represent the core functionality of the platform, and the integration of data across the functional components to manage and store dynamic user profile processing is described as a separate functionality. This will enable the mass production of user-driven driver behavior-related decisions and provide a basis for creating the next-generation of driver behavior risk prediction. The DDP components and functions describe the recommender system processing functionality using user profile content processing and management, and are integrated into driver behavior analysis in hybrid learning recommendation processing using machine learning techniques. The DDP intends to use the user context in recommendations and provides adaptive contents that are reorganized according to the recommendation platform environments. This primarily comprises driver behaviors based on historical accident and violation record standard options, which include functional and nonfunctional requirements, further divided into an automatic configuration of behavior risk assessment and extraction of detailed dynamic profile elements.

Therefore, all four systems work together to prevent accidents and generate early warning alerts to the vehicles to provide safety services. Towards this aim, this work presents multiple contributions which are explained as follows:

- The user profile contents processing and management system includes functions that allow the user to register and create a new profile, search and manage profile information, and process feedback into a system database. The user profile includes basic user information and external situational information such as location, time, etc. In the profile analysis, there is a process for storing profiles for users and providing patterns to each module which is linked with the profile module’s interface, shown in Figure 5.
- The recommendation processing and services model consists of three main functions, which are user and contents profile filtering, hybrid recommendations, and machine learning processing to provide content to users through integration of driver behaviors and the dynamic user’s profile contextual information. When a recommended list of contents is provided to users, prioritization of contents is made considering the user’s feedback as reflected and provided in the contents, and then the contents can be served according to the determined contents. The mobile application is an application that shows the contents to users, and it is also a module that transmits user profile information onto the platform.
- The driver behaviors risk analysis process uses rule-based classification, the risk matrix classifies the parameters using the Saudi Traffic Points System Regulation (STPSR). The rules for deciding the number of points calculated for every violation/accident of traffic regulation risk likelihood are divided into 4 risk score levels, which are high, medium, low risk, and no risk drivers, determined based on driver historical violation/accident records to predict current and future driver risk scores using the STPSR system. To predict the driver’s risk score we used this equation: human errors (HE) + violation group (VG) = prediction driver risk score (PDRS) during a 12 month period, as shown in Table 1.
- Maintaining a single profile for the user preserves a consistent experience by giving the user an intuitive management system which is allowed by the profile management and feedback processing. Exchanging and managing refer to extracting driver risk behavior and user profile elements by analyzing profile contents, and then linking them with data storage. The content manager also automatically constructs contents generated by the STPSR index violation/accident, the DDP element analysis management, and the general effect/efficacy information of the driver risk behavior, and then stores them in the database. The effect of the information is generated from the feedback of the user and the profile information reflecting information stored in the profile databased. However, the first priority of this paper is to enhance the quality of road safety for the individual in the field of intelligent traffic recommendation services, which can be done by integrating the user and vehicle information, violation and accident records, and driver habits into integrated customized contents, which in turn requires hybrid learning recommendation processing [21].

## 4. Deep Learning Modeling and Data Classification for DDP

**Figure 6:**
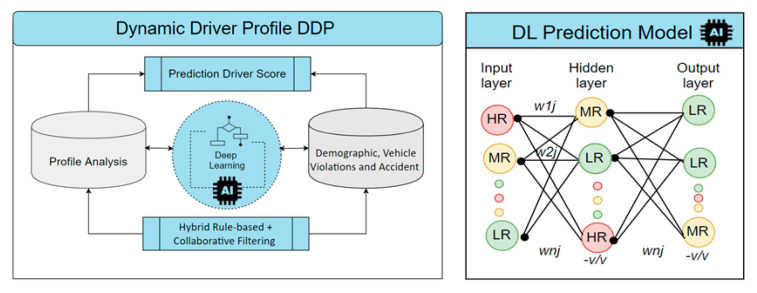
Deep learning prediction model.

The core idea behind DDP processing is to apply deep learning processing by computing the similarity index as pre-processing between dynamic users’ profile and driver risk score content shown in Figure 6. Normalization of the input values is often performed to improve the training process speed. Typically the values are normalized between −1.0 and 1.0 and are encoded in such a manner so that each domain value is evaluated to one input unit.

Repetitive training of the network is a typical scenario in machine learning-based systems as this helps in achieving the best possible accuracy of a given model, and this can be done by choosing different network architectures and/or modifying the initial weights. Algorithm 1 presents the pseudo code for the proposed DDP hybrid learning recommendation processing. This was used in calculation for a comparison of the recommendation list (rec_ score_list) with the proposed, hybrid recommendation system based on DDP model algorithm.
**Algorithm 1** Driver Accident/Violation Classification System1: X ← User List2: Y ← List of Varying User Lists3: neuralnet.Create()4: neuralnet.addLayer15: neuralnet.addLayer26: neuralnet.addLayer37: *loss_msc_*.Compute()8: epoch = 10009: **for** i in epoch **do**10:     neuralnet.fit(X, Y)11: **return** neuralnet.predictedval()12: **function SHOW ACCIDENT(ID, year)**13:     **if** *num_accidents_* > 0 **then**14:         *print*(ID, date, Vehicle No., Penalty Pts, Mistake)15: function **SHOW VIOLATIONS(ID, year)**16:     **if**
*num_violations_* > 0 **then**17:         *print*(ID, date of stop, Vehicle No., Penalty Pts)18: **function SHOW DRIVER(ID)**19:     *print*(ID, Age, Gender, Nationality)20: **function EVALUATE DRIVER(ID, year, accidentdata, violationdata, driverdata)**21:     **if** *totalpoints* <= 4 **then**22:         *print(*No Risk*)*23:     **else if**
*totalpoints* <= 25 **then**24:         *print(*Low Risk*)*25:     **else if**
*totalpoints* <= 50 **then**26:         *print(*Medium Risk*)*27:     **else if** *totalpoints* > 50 **then**28:         *print*(High Risk)29: *print*(Total Penalty Points)


The proposed method has been implemented in a use case in the Saudi Arabian transportation system on three helper functions, to find the best drivers based on accident patterns and frequency, and the similarity function to calculate and return the similarity matrix in Table 2, to explain the python library’s function description, which uses the python environment and hybrid recommendation methods to enable an automatic intervention based on driver risk level. It also helps to solve the challenges in the recommender system, such as cold-starts for new users and low-accuracy in the data, to some extent, which are inherent in current recommendation engines.

### 4.1. Generating and Preprocessing DDP Dataset

In this section we explain the data-generating and preprocessing function that adapts dataset preparation to the process of transforming raw data so that the dataset can be analyzed and run by machine learning algorithms to uncover insights into predictions. This function is also used to create a model in machine learning by acquiring input datasets, preparing the data, defining the features, training and testing the model, and predicting new data output.

To generating the DDP dataset to implantation and evolution, the results in the dataset include driver’s information, registration of the vehicle, and violations and accident records; the purpose of this data is to identify driver risk behaviors. In addition, we used a dataset that includes accidents in Saudi Arabia from 2015 to 2018. Then, we created a dynamic driver profile that includes GPS trajectories for multiple driver trips in Saudi Arabia and their events, which are identified using INS navigation devices. We will depend on the count of risky actions performed by drivers, such harsh acceleration, harsh turns, and harsh brakes. The more of these risky events that occurred, the riskier the driver was determined to be. Table 2 shows the four generated driver dataset records containing user (driver) information, vehicle information, and functional or behavioral events recorded in the event column in the Table 3, in addition to the metadata as recorded in the Table 4.

### 4.2. Analysis of Accidents in Saudi Arabia

**Figure 7:**
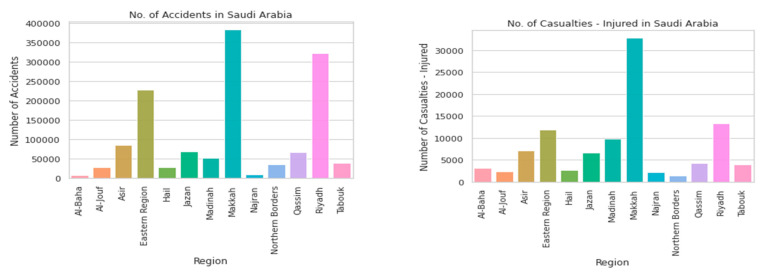
Number of accident and injuries in Saudi Arabia.

According to a recent estimate, there are more than 6 million cars on the road in KSA. Road traffic injuries (RTIs) are a leading cause of mortality and negatively affect the quality of life in Saudi Arabia, as shown in Figure 7. The high number of casualties due to RTIs in Saudi Arabia is the highest among high-income countries and is also considered the primary reason behind the deaths for males in the age range of 16–30 years old. Therefore, a major goal towards reaching the top 5 most livable countries is to decrease traffic deaths. A research study and analysis indicates that accident/injury in Mecca city is very high in comparison to other cities in Saudi Arabia, which needs to be studied to provide a solution to the factors that contribute to Mecca having the highest number of accidents [4].

### 4.3. Predicting Driver Risk Classification

The basic idea of the model fitting process is to use drivers’ two year prior violation and accidents records (using prior features to predict their future driving risk based on two years). Table 5 shows the driving behavior evolution parameters based on record violation and accidents, then the classification of drivers who are sorted into the categories of HR, MR, or LR. The model was established to predict drivers’ risk behaviors based on their record from the previous two years. In measuring driving risk, if the driver varies over time the driver can be defined as HR in one observation period (e.g., 2015–2016) but LR in another period (e.g., 2015–2017) based on their accident records to implement DDP that evaluates drivers and identifies which one is a safe driver and which one is a risky driver. Here we used a dataset that includes accident and violation historical records for multiple drivers in Saudi Arabia and their risk scores that were identified using STPSR Risk Metrics.

**Figure 8:**
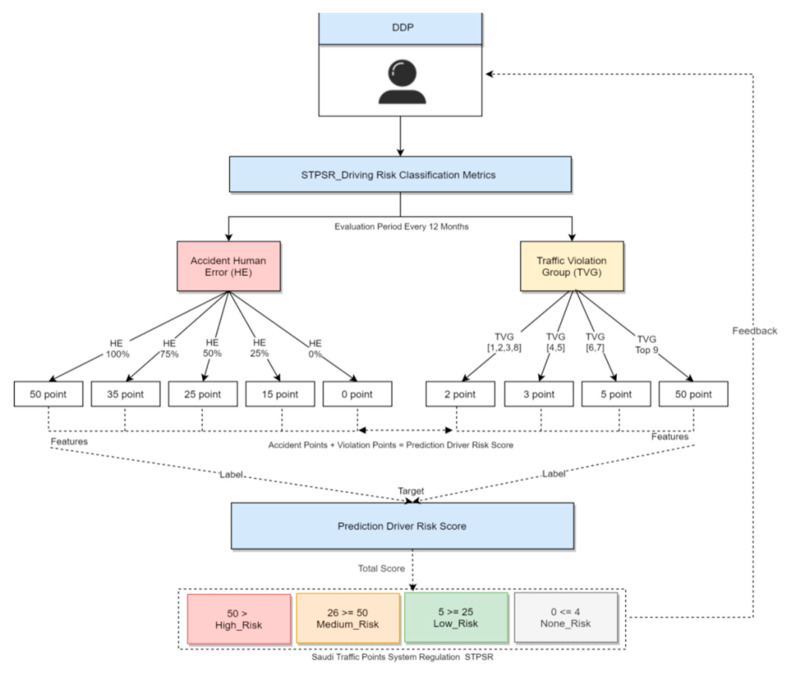
Driver risk classification based on STPSR model risk.

We will depend on the count of the risky actions performed by drivers, like the violation/accident records, to predict that the more these risky actions occurred, the riskier the driver. Figure 8 shows the proposed solution to the driver risk criteria that we used in the classification approaches, but it can be modified based on the Saudi traffic regulation system using the driver risk score classification and hybrid recommendation system prediction methods. 

### 4.4. Accident and Violation Classification

**Figure 9:**
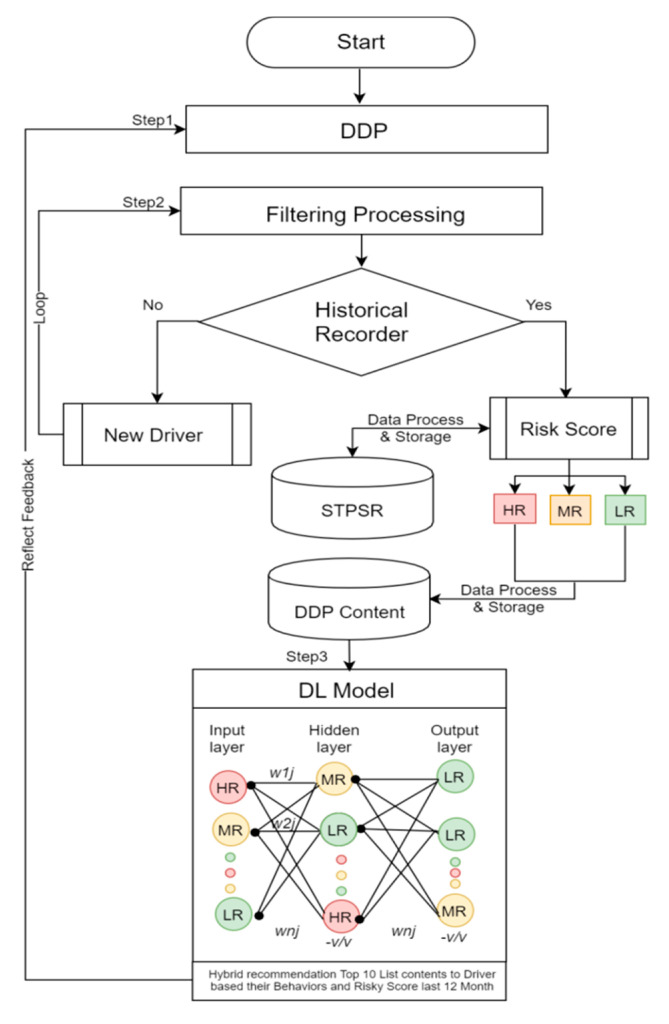
DL prediction model for driver risk classification.

The accident and violation classification mechanism is considered to convert human error “driver-mistake” percentages to penalty points. For example, a mistake of 13% will be converted to 0 points since its within scenario 5, Grouping violations based on the Saudi violation lists, based on STPSR category 10, which is a special category which groups the highest penalties (50 pts). The proposed hybrid recommendation system has considered machine learning to analyze the risky driver score model which is shown in Figure 9. The basic idea of the model fitting process is to use drivers’ Q3 prior violation and accidents records each year, as shown in Table 4 and Table 5, using prior features to predict their future driving risk based on the last 5 years. Driving behavior evolution parameters based on record violation and accidents then classify the driver’s risk level. The machine learning model predicts the driver’s risk behaviors based on their behaviors in the previous 5 years.

### 4.5. Measure Driving Risk: Driver Varies over Time

**Figure 10:**
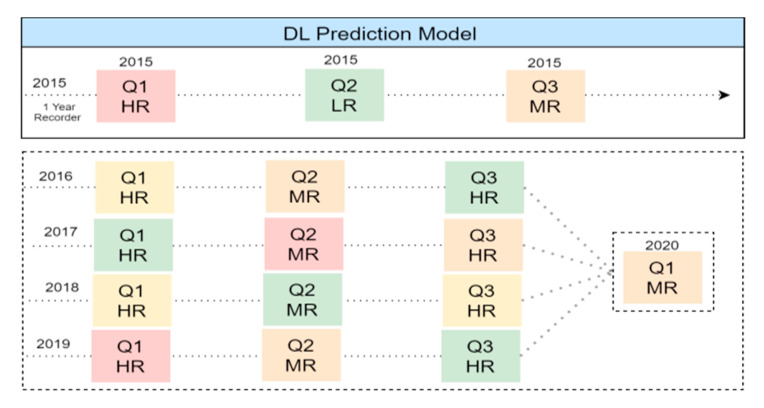
The visualization of top driver’s violations records.

A driver can be defined as HR in one observation period (e.g., 2018–2019) but LR in another period (e.g., 2017–2018) based on their accident records. The driver risk score will take the average of the previous 5 years’ total points to predict driving risk factors based on the Saudi traffic points system using historical violation and accident records shown in Figure 10.

Collaborative filtering (CF) is a recommendation algorithm that can utilize the previous user’s rating to identify the new user’s similarity based on their behavior, and thus predict the new user’s preferences on further items. It works as a one-to-one matching algorithm. In this algorithm, other users’ data can be used to provide an appropriate prediction of the current users’ preferences. The algorithm assumes that the new user does not have any historical actions, besides the rating user-user matrix process on the similarity function sim:(user1 × user2), CF: u/u calculated using the Euclidean distance similarity at Equation (1) and shown in Table 6.

(1) $$\mathrm{Euclidean}\;\mathrm{Distance}\;\mathrm{Similarity}=\sqrt{\sum_{n=1}^{n}{\left(x_{i}-y_{i}\right)}^{2}}.$$

From these results we can see that driver risk evolution loops the start time every 12 month from 1 January to 31 December for the risk records of either accident or violation. Each driver’s recorded risk score become zero and a new loop starts every year. For calculating the driver risk score we used a parallel recorder for (each 1 year) + (average of 5 years) in the ML model prediction; using the 5 year average, then classifying driver risk score results. This proposed algorithm processing and filtering of dynamic context extraction and context reasoning combine the context information from smartphone sensors and user profile and preferences to improve the efficiency and usability of the recommendation by Equation (2) and Table 7.

(2) $$\mathrm{similarity},\;\mathrm{sim}\left(uidi,\mathrm{tp}i\right)=\sqrt{\frac{\sum_{i=1}^{n}{\left(uidi-\mathrm{tp}i\right)}^{2}\times uidi}{\sum_{i=1}^{n}uidi}}$$

where: u*i*d*i*: user id & tpi: total points risk score id, calculated using the Cosine.

## 5. Performance and Evaluation

In this section, the experimenting and evaluation phase steps are presented, which were calculated for a comparison of the recommendation list (rec_ score_list) with the proposed hybrid recommendation system based on the DDP model. The experiments were conducted to verify the performance of the proposed DDP. In this experiment, rec_score_list stored the actual values for each datapoint. The error values were calculated and compared using a variety of minimum error computation functions. A low error value for the predicted value signifies a higher accuracy of being close to the actual correct value. In contrast, a higher error value signifies that the predicted value becomes less important for the recommendation outputs. The experiments proceeded in the following environment, to be explained in the next section.

### 5.1. Experimental Environment

We used python language to process the data analysis and prediction. These experiments were performed using several python libraries. The algorithm uses three matrix error models, namely MAE, MAPE, and MSE, to evaluate the recommendation results. First, we started with the classification function, which was used to classify a driver, given driver ID. To define the accident/violation classification function based on driver ID we applied the hybrid score risk list. The model used mean squared error as loss function, Adam as optimizer, and accuracy and mean absolute error (MAE) as metrics during training. The model was run for 1000 epochs with 15% data as validation data. Next, there was a function for showing accident data, violation data for a particular year, and a function for printing driver details. Finally, the driver was classified using the penalty points from accident data and violation data.

**Figure 11:**
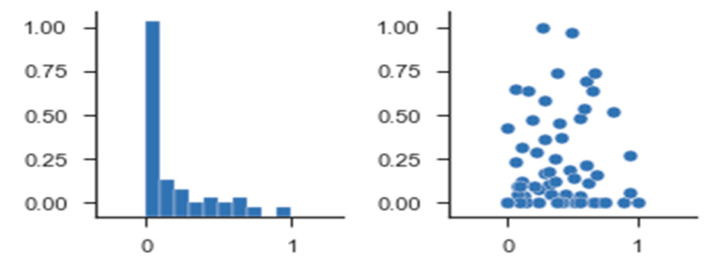
The visualization of top driver’s accident records.

**Figure 12:**
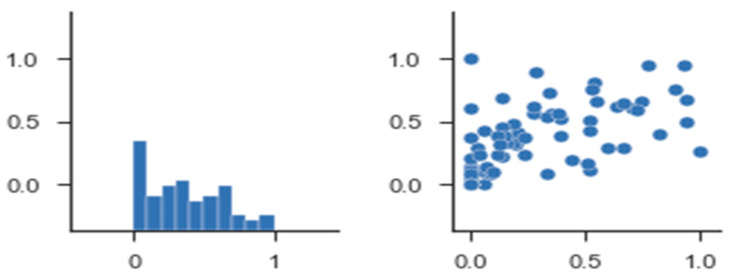
Performance analysis of predication accuracy.

From the experimental algorithm, we have tried to obtain the top 5 drivers that have the greatest number of violations in our dataset, obtaining the data of the top driver with most violations, driver ID (A53160534/40, A51132989/40, A35359040/40, A30901291/39, A51406120/39) and accidents, total number of violations per year for selected driver, and total number of accidents per year for selected driver. Next we prepared data for the visualization of violations, visualizing the violations and accident data for the top driver, ID: A51132989. Violation records from the years 2015 to 2020 are shown in Figure 11 and Figure 12.

Moreover, we can see in the Figure 11 and Figure 12, where the top driver has recorded 40 violations and 18 accidents in total from 2015 to 2020; however in the last 2 years (2018–2019) there were 19 violations and 6 accidents, the total number of medium risk violations made in the last 2 years was 6 violations, the total number of low risk violations made in the last 2 years was 7 violations, the total number of high risk accidents occurring in the last 2 years was 0 accidents, the total number of medium risk accidents occurring in the last 2 years was 2 accidents, and the total number of low risk accidents occurring in the last 2 years was 5 accidents. Based on the last few calculations we can classify the driver based on the total count of violations and accidents sorted into the categories of of high, medium, and low risk events occurring in the last 2 years. We can see that the driver was involved in 19 violations and 7 accidents in the last 2 years. We can say that the total number of events was 26. There were 6 high risk violations, while there were no high risk accidents, and that means that there were 6 high risk events occurring in the last 2 years. There were 6 medium risk violations, and there were 2 medium risk accidents, and that means that there were 8 medium risk events occurring in the last 2 years. There were 7 low risk violations, while there were 5 low risk accidents, and that means that there were 12 low risk events occurring in the last 2 years. From this we can calculate the percentage of events in each category for the driver as high risk category 6/26 × 100 = 23%, medium risk category 8/26 × 100 = 31%, and low risk category 12/26 × 100 = 46%. From these calculations we can say that because 46% of events were low risk, and because the percentage is lower than half of the total, so we should treat him as medium risk and give him all of the instructions that should follow when a medium risk driver is identified. Also we can make the percentage increase or decrease using the layer recording the previous 2 years; if by this criteria the percentage of low risk events is higher than the current percentage then the driver’s rate will decrease because their behaviors has become more reckless, and also because the driver will be sent warnings and advice based on the kind of violations made or accidents occurring. If the low risk percentage of the previous layer is less than the current layer, that means that the driver’s behaviors are becoming less risky and their rate should be increased and they should be rewarded to encourage the driver to get better.

### 5.2. Model Performance Evaluation

The models’ performance was evaluated using several error calculation formulae, shown in Table 8.

**Figure 13:**
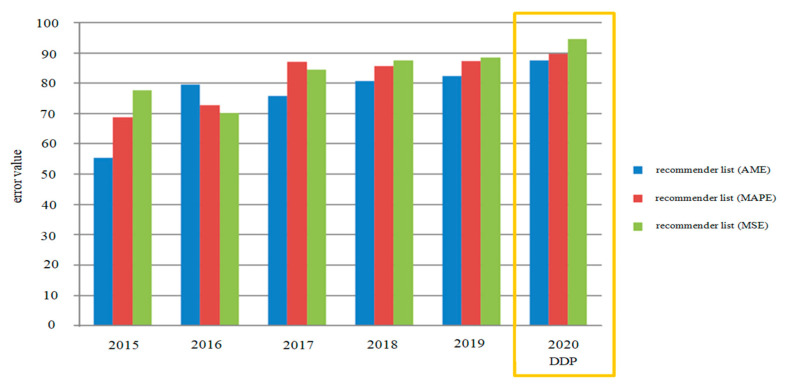
Performance analysis of predication accuracy.

The error values calculated using MAE were calculated as shown in Table 8. Prediction accuracy was a formal model to compute the aggregated error values using a combination of MAE, MAPE, and MSE error functions over the period of 2015 to 2020. The calculation results of error numbers using MSE, showing the lowest error value that can solve cold-start and scalability issues in MAE by 0.85/0.56 and MSE about 5.23/4.32, are shown in Table 9. Figure 13 provides a way to calculate error values of MAE, MAPE, and MSE over 5 years from 2015 to 2020, comparing the proposed deep learning DDP model to predictions for 2020. Unlike MAE, it squares the values to weigh in on the higher error values. Doing so penalizes the higher error scores compared to the lower error scores; according to the rec_list results, the least error value was generated when good results were shown with MAE.

## 6. Conclusions

In this paper we have proposed a novel approach for continuous monitoring of driver, motion, and road analysis to prevent accidents, and described a use case of its implementation. We have achieved the motion prediction by using deep learning methods. In this paper, we have introduced a dynamic driver profile (DDP) for behavior risk prediction using recommendation based on deep learning methods to enable automatic interventions for the safety of the driver, which works upon three major sets of attributes that predict the motion accurately. First, we propose the high level architecture for the DDP and components forming the overall architecture. Then, the interactive deep learning process design is illustrated. Training for the prediction models is expected by way of the participant’s driver historical accident and violation records, and deep learning recommendation based on driver feedback and performance. Finally, some preliminary scenarios and experiment results are shown, and a discussion on future directions is presented. We envisage the proposed system to be digitally implemented and behaviorally designed to predict driver risk behavior and minimize the numbers of high-risk drivers. The road analysis was constructed as a Bayesian dependency graph, which is updated frequently to maintain a solid record of road conditions, and early warning alerts are generated for all risky drivers that could improve the overall recommendation performance. A feedback-based update is also presented to improve the accuracy of the prevention platform. For the future development of the recommendation system, the perceptron DDP learning model used for deep learning algorithms and other machine learning techniques such as reinforcement learning can be used to improve the current research and overcome limitations [22]. In addition, experiments on recommendation accuracy and error frequency are required that can improve the scalability and latency performance.

## Figures and Tables

**Table 1:** Driver risk metrics based on the STPSR system.

Accident Points System Human Errors (HE)	Traffic Violation Groups (VG)	Predication Driver Risk Score (PDRS) Every 12 Month	
HE 100% = 50 points HE 75% = 35 points HE 50% = 25 points HE 25% = 15 points HE 0% = 0 points	VG1,2,3,8 = 2 points VG4,5 = 3 points VG6,7 = 5 points VG9_Top Risk = 50 points	Total Points	Risk Group
		0 ≤ 4	None
		5 ≥ 25	Low
		26 ≥ 50	Med
		50>	High

**Table 2:** Generated driver’s dataset.

	National_ID	Age	Gender	License	Nationality
0	90928938	43	M	Yes	Indian
1	83545661	74	F	Yes	Pakistani
2	07316072	89	M	Expired	SAUDI Arabs
3	07677590	34	M	Expired	Pakistani
4	22584531	23	F	Yes	Indian

**Table 3:** Generated drivers and vehicle dataset.

Driver ID		Vehicle	Vehicle_Number	Vehicle Type	Make	Model	Year	Color
0	737	02-Automobile-HONDA-ACCORD-1990.0-BLUE	CXP 104	2	Autmobile HONDA	ACCORD	1990.0	BLUE
1	771	02-Automobile-HONDA-FIT-2010.0-SILVER	MNZ 788	2	Autmobile HONDA	FIT	2010.0	SILVER
2	2236	02-Automobile-BMW-2S-2007.0-SILVER	OBV 546	2	Autmobile BMW	2S	2007.0	SILVER
3	4046	02-Automobile-HONDA-ACCORD-2013.0-BLACK	ALG 828	2	Autmobile HONDA	ACCORD	2013.0	BLACK
4	4445	02-Automobile-HONDA-ACCORD-1998.0-GREEN	DDL 041	2	Autmobile HONDA	ACCORD	1998.0	GREEN

**Table 4:** Event based driver’s dataset.

Driver ID	Event	Latitude	Longitude	Speed (km/h)	Time Stamp (TS)
0	Timed Event	34.186631	−118.088102	64.0	2020-11-01 00:00:02.430
0	Distance Event	34.186060	−118.089241	53.0	2020-11-01 00:00:05.600
0	Distance Event	34.186408	−118.089560	34.0	2020-11-01 00:00:13.640
0	Distance Event	34.187479	−118.088915	33.0	2020-11-01 00:00:26.070
0	Distance Event	34.188665	−118.086459	32.0	2020-11-01 00:00:35.090
0	Distance Event	34.188171	−118.087279	47.0	2020-11-01 00:00:46.330
0	Distance Event	34.189409	−118.086420	24.0	2020-11-01 00:00:55.320
0	Timed Event	34.188765	−118.086086	35.0	2020-11-01 00:01:02.770
0	Distance Event	34.189057	−118.085112	43.0	2020-11-01 00:01:06.610
0	Distance Event	34.190146	−118.083935	48.0	2020-11-01 00:01:14.530

**Table 5:** DDP deep learning model parameters.

Parameters	Explain
HR, MR.LR	Driver ID/risk criteria _initial input unityi
Wnj	Weight_increased, decreased
−*v*/*v*	BiasValue = (−1.0 and 1.0)
knn	k-nearest neighbors learning methods

**Table 6:** Driver risk score over 5 years processing by CF user/user similarity matrix.

USER/Quarters	Year 1			Year 2			Year 3			Year 4			Year 5		
	Q1	Q2	Q3	Q1	Q2	Q3	Q1	Q2	Q3	Q1	Q2	03	Q1	Q2	Q3
U1	SP_LR	16P_LR	13P_LR	2P_NR	0P_NR	4P_NR	90P_HR	53P_HR	79P_HR	27P_MR	35P_MR	50P_MR	63P_HR	70P_HR	83P_HR
U2	0P_NR	0P_NR	2P_NR	BP_LR	12P_LR	20P_LR	66P_HR	83P_HR	54P_HR	72P_HR	55P_HR	B0P_HR	22P_MR	30P_MR	48P_MR
U3	66P_HR	52P_HR	72P_HR	49P_MR	36P_MR	28P_MR	25P_LR	13P_LR	6P_LR	SSP_HR	62P_HR	52P_HR	86P_HR	90P_HR	95P_HR
U4	28P_MR	40P_MR	38P_MR	SP_LR	12P_LR	24P_LR	2P_NR	2P_NR	2P_NR	86P_HR	60P_HR	75P_HR	36P_MR	48P_MR	28P_MR
U5	70P_HR	75P_HR	SSP_HR	4P_NR	4P_NR	4P_NR	47P_MR	28P_MR	45P_MR	SP_LR	24P_LR	16P_LR	0P_NR	2P_NR	0P_NR

**Table 7:** Driver risk score average over 5 years.

USER	Y1	Y2	Y3	Y4	Y5	Total Points	5 Years Average	Risk Score Results
U1	24P_LR	2P_NR	53P_HR	50P_MR	53P_HR	182	36.4	Medium-Risk
U2	4P_NR	15P_LR	75P_HR	83P_HR	26P_MR	203	40.6	Medium-Risk
U3	59P_HR	50P_MR	25P_LR	83P_HR	53P_HR	270	54.0	High-Risk
U4	37P_MR	6P_LR	2P_NR	90P_HR	32P_MR	167	33.4	Medium-Risk
U5	65P_H R	4P_NR	40P_MR	10P_LR	0P_NR	119	23.8	Low-Risk

**Table 8:** Formulas of MAE, MAPE, and MSE evaluation criteria.

Evaluation Criteria	Formula	Functional
MAE	$\frac{1}{n}\sum_{1}^{n}\left|d_{i}-{\hat{d}}_{i}\right|$	Mean absolute error is less sensitive to the outlier values and cannot solve the scalability issues.
MAPE	$\frac{100}{n}\sum_{1}^{n}\frac{d_{i}-{\hat{d}}_{i}}{d_{i}}$	Mean absolute percentage error works in a similar manner to MAE but is normalized by true observation. It measures the recommendation accuracy in percentage.
MSE	$\frac{1}{n}\sum_{1}^{n}{\left(d_{i}-{\hat{d}}_{i}\right)}^{2}$	Mean squared error is capable of solving the cold-start and scalability issues as it works by measuring the combination of both bias and variance.

**Table 9:** Performance analysis of average error value and predication accuracy.

Comparison of the Proposed DPP Predication Model During 5-Years Top Recommendation List of Average Error Results						DDP
Model	2015	2016	2017	2018	2019	2020 Prediction
rec_list (MAE)	54.78/55.33	79.5/79.6	75.17/75.72	80.22/82.77	82.379/83.443	87.11/88.41
rec_list (MAPE)	68.12/68.67	72.25/72.81	86.45/87	85.14/86.69	87.329/88.384	89.16/90.18
rec_list (MSE)	77.9/77.64	69.65/70.2	84.3/84.58	87.6/88.15	88.369/89.424	94.16/95.12

## Data Availability

All data and models used during this research are confidencial in nature and may only be provided with restrictions.
